# Non-invasive Respiratory Support in COVID-19: A Narrative Review

**DOI:** 10.3389/fmed.2021.788190

**Published:** 2022-01-04

**Authors:** Manel Luján, Javier Sayas, Olga Mediano, Carlos Egea

**Affiliations:** ^1^Pneumology Service, Hospital Universitari Parc Taulí, Sabadell, Spain; ^2^Centro de Investigacion Biomédica en Red (CIBERES), Madrid, Spain; ^3^Pneumology Service, Hospital Universitario 12 de Octubre, Madrid, Spain; ^4^Pneumology Department, Hospital Universitario de Guadalajara, Guadalajara, Spain; ^5^Hospital Universitario de Araba, Universidad País Vasco, Vitoria Gasteiz, Spain

**Keywords:** CPAP, high flow oxygen therapy, non-invasive ventilation, acute distress respiratory syndrome, prone position

## Abstract

Acute respiratory failure secondary to COVID-19 pneumonia may require a variety of non-pharmacological strategies in addition to oxygen therapy to avoid endotracheal intubation. The response to all these strategies, which include high nasal flow, continuous positive pressure, non-invasive ventilation, or even prone positioning in awake patients, can be highly variable depending on the predominant phenotypic involvement. Deciding when to replace conventional oxygen therapy with non-invasive respiratory support, which to choose, the role of combined methods, definitions, and attitudes toward treatment failure, and improved case improvement procedures are directly relevant clinical questions for the daily care of critically ill COVID-19 patients. The experience accumulated after more than a year of the pandemic should lead to developing recommendations that give answers to all these questions.

## Introduction

Severe acute respiratory syndrome caused by coronavirus-2 (SARS-CoV-2) emerged at the end of 2019 in Wuhan, China, resulting in an ongoing global respiratory illness pandemic, named Coronavirus Disease 2019 (COVID-19) (1). COVID-19 has a wide spectrum of clinical severity, ranging from asymptomatic to critically ill patients, and ultimately death. The most common feature of severe COVID-19 disease is acute hypoxemic respiratory failure (ARF) requiring oxygen and ventilatory support, and it has been reported that about 5% of the infected patients develop a life-threatening clinical picture (2).

The characteristic pattern of severe disease due to COVID-19 is bilateral pneumonia matching the criteria of acute respiratory distress (ARDS), although some authors defend that there are pathophysiological differences between classic distress and that associated with COVID-19, the so-called C-ARDS (3). C-ARDS is a heterogeneous entity from a clinical point of view, a fact that it shares with ARDS. An attempt has even been made to classify it according to lung mechanics into two different phenotypes. In the first phenotype (L, related to low elastance, low lung weight, and low recruitability), ventilation-perfusion mismatch would predominate, with relatively preserved pulmonary mechanics (compliance around 40-50 ml/cm H2O). In L phenotype, the main pathophysiological phenomena would be the lack of regulation of the pulmonary vasculature, with loss of the hypoxic vasoconstriction mechanism, inflammatory hyperemia of the collapsed areas, and hypoperfusion of the peripheral regions. This phenotype usually corresponds to an early phase of the disease. The second phenotype (or H phenotype) would be like classic ARDS, with high elastance, recruitability, and collapse of dependent areas, often corresponding to a later phase of the disease. In both cases, the presence of thrombotic phenomena at the level of the pulmonary micro and macrovasculature can further aggravate the ventilation perfusion mismatch (4, 5). Interestingly, despite severe hypoxemia, the infected patients often present with less dyspnea than expected (the so-called “happy hypoxemia” or “silent hypoxemia”), probably due to the preserved pulmonary mechanics, as demonstrated by Chiumello et al. in a comparative study about the features of C-ARDS and non-COVID ARDS (6). Another physiopathological explanation for this “happy hypoxemia” has been proposed by Jounieaux et al. (7). As stated by these authors, the presence of right-to left intrapulmonary shunt induces hypoxemia, leading to an increase in minute ventilation. This increase in minute ventilation may not be enough to increase SpO_2_ (as oxygenation increase may be blunted by shunt effect) but may lead to hypocapnia. Hypocapnia has been proven to be a strong driver to decrease dyspnea. For these reasons, the acronym “AVDS” (acute vascular distress syndrome) has been proposed by these authors (7, 8). Other authors have proposed several other mechanisms to explain this silent hypoxemia, such as fever (shifting to the right the oxygen dissociation curve), age, some comorbidities, or pulsioxymetry sampling limitations (9). The underlying vascular abnormalities have also been demonstrated both in autopsy series and in radiological studies (10, 11).

Orotracheal intubation and mechanical ventilation, with protective strategies to avoid aggravating lung injury, have been the main ventilatory support treatments for conventional ARDS, until resolution of the causal process (12). However, in COVID-19, the large number of patients who were infected simultaneously caused the demand for mechanical ventilation to be widely exceeded. In this overwhelming setting, many patients with COVID-19 and ARF required non-invasive respiratory support (NIRS), beyond conventional oxygen therapy (COT). However, there are no unitary protocols regarding when NIRS should be started, what type of support to use, its duration, failure criteria, and treatment withdrawal.

There is a wide range of experience in the use of different non-invasive respiratory support modalities that may need to be reviewed. Non-invasive ventilation (NIV) or CPAP has been used to avoid intubation in hypoxemic patients for more than 20 years (13). Throughout the last decade, another form of NIRS, the high-flow oxygen therapy (HFOT), has gained popularity. It started as a tool mostly used in pediatrics, and jumped to adult use with a growing body of evidence. Nowadays, its use has expanded in an exponential way (14).

For the current narrative review, a PubMed search was performed with the following MeSH headings and search strategy: ((((((“Continuous Positive Airway Pressure”[Mesh]) OR “Respiratory Therapy”[Mesh]) or “Noninvasive Ventilation”[Mesh]) OR “Intermittent Positive-Pressure Ventilation”[Mesh]) OR “Positive-Pressure Respiration”[Mesh]) OR “high flow nasal cannula” [Mesh] OR “high flow oxygen therapy” ([Mesh]) AND ((“COVID-19”[Mesh]) OR (“SARS-CoV-2”[Mesh]))))) AND TREATMENT[filter]. Search was restricted to “Clinical trials,” “Meta-Analysis,” “Randomized Controlled Trial,” “Review,” and “Systematic Review.”

**Figure 1:**
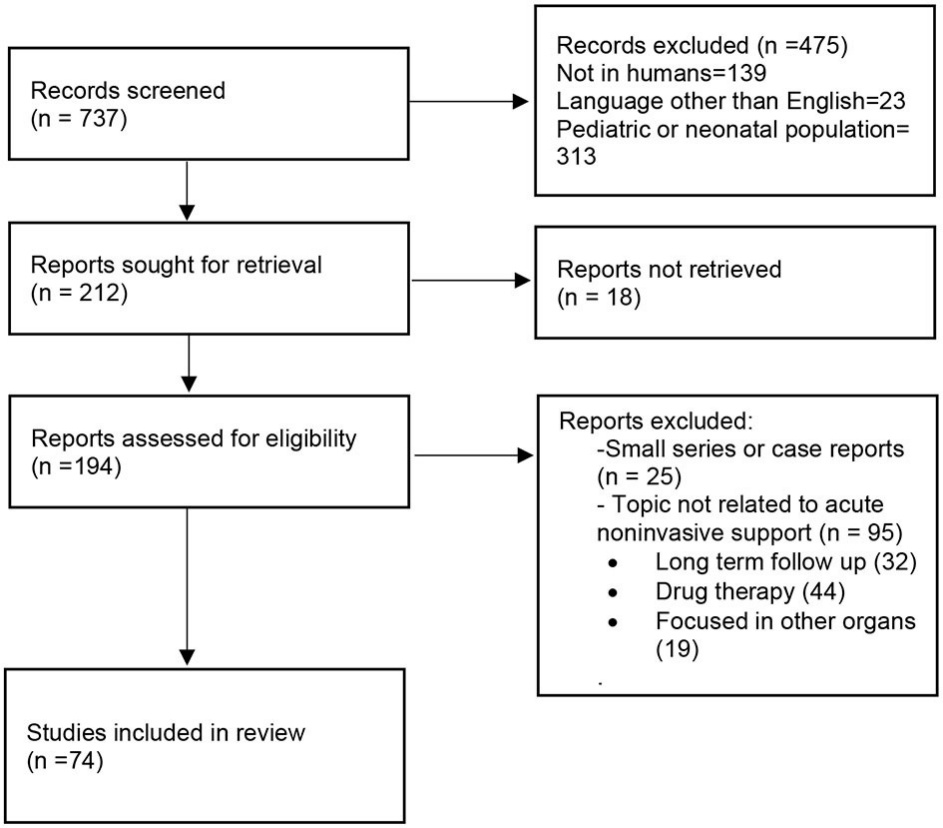
PRISMA-based flowchart for the selection of references.

With that search strategy 737 results were screened, and 212 results were finally retrieved. As we did not intend to perform a meta-analysis, we refined the search eliminating case reports or other trials not related to non-invasive respiratory support. Figure 1 shows the PRISMA-based flowchart (15) for the selection of references.

## The Role of Non-invasive Ventilatory Support in Covid-19

Traditionally, in hypoxemic ARF in acute respiratory distress, one of the main concerns is the increased mortality associated with intubation delay. Thus, NIV has been widely questioned as a support method. In a recent international observational study that included 2,813 patients with acute respiratory distress (ARDS), those initially treated with NIV (15%) and severe hypoxemia (PaO_2_/FiO_2_ < 150 mm Hg) had higher mortality (36.2%) than those ventilated invasively (24.7%) (16). In contrast, HFOT has emerged as a non-invasive strategy for avoiding intubation and invasive ventilation. In the FLORALI study (17), although the result for the primary endpoint (intubation rate) was negative, mortality and the number of days free of mechanical ventilation were significantly lower in the group treated with HFOT. In the subgroup study, the authors found a significant reduction in the intubation rate in patients with more severe hypoxemia (PaO_2_/FiO_2_ < 200).

Based on these previous experiences in hypoxemic ARF and NIRS, as the first phase of the COVID-19 epidemic overflowed, several guidelines from different countries recommended early intubation of critically ill patients with COVID-19 and ARF, also as a means of protecting healthcare workers from cross-infection (18, 19).

One of the main reasons stated for recommending early intubation in patients with COVID and ARF would be the fact that the use of NIRS techniques delays rather than prevents intubation. This delay, while maintaining spontaneous respiratory pattern with tachypnea and high tidal volume, may lead to the worsening of the so-called patient self-induced lung injury (P-SILI). P-SILI has been linked to various pathophysiological phenomena: (a) increased effort, both inspiratory and expiratory, can lead to an increase in transpulmonary pressure (stress) and strain (increase in volume with respect to its baseline value). The intensity of the inspiratory effort has been correlated as a surrogate of the neural drive associated with relapse in patients with COVID 19 (20); (b) inhomogeneity in gas distribution, with areas with different time constants and intrapulmonary gas redistribution between them (pendelluft phenomenon); and (c) changes in pulmonary perfusion (21).

On the other hand, the defenders of NIRS techniques (high nasal flow and positive pressure, either continuous positive pressure—CPAP- or bilevel) argue that they can avoid unnecessary endotracheal intubations and that the liberal use of invasive ventilation and its associated consequences (muscular atrophy and ventilation associated infections) may lead to increased mortality.

The experience in the use of NIRS in COVID-19 comes mainly from retrospective observational studies, with extremely variable failure rates, ranging between 20 and 60%, and biased populations (i.e., age selected, Intensive care Unit—ICU—or ward environments). A meta-analysis about non-invasive ventilatory support (HFOT was excluded) as a therapeutic option outside the Intensive Care Units included 3,377 patients. Overall mortality was 38%, although it is possible to distinguish the group of patients without therapeutic limitation (19%) from that of patients with orders of no intubation (72%). Mortality in patients with NIV failure who were ultimately intubated was 45% (22).

There are no prospective studies focused on the outcome of patients with direct intubation vs. a previous trial with non-invasive support. A recent meta-analysis that included 8,944 patients showed no benefit of early intubation compared to intubation delayed more than 24 h after admission to the ICU, neither in mortality nor in days of mechanical ventilation. Mortality was also not significant in patients who received treatment with high nasal flow or non-invasive ventilation compared to those who did not receive such treatment before intubation (23).

Therefore, with the available data, the use of NIRS does not seem to lead to a worse prognosis when compared with direct orotracheal intubation.

## Beyond Oxygen Therapy. When to Start Non-invasive Ventilatory Support

Conventional oxygen therapy has clearly been the main supportive technique in ARF secondary to COVID-19 (24). However, in a percentage of patients this technique may not be enough to ensure proper oxygenation, and it has been necessary to choose between available NIRS techniques: high nasal flow therapy, treatment with positive pressure-CPAP, or bilevel pressure systems (25). A paramount issue is the timing of starting a NIRS. Both positive pressure systems and high nasal flow have a certain unloading effect on the inspiratory musculature, while improving pulmonary gas exchange. On the other hand, the efforts made by the patient in spontaneous ventilation in the presence of respiratory failure can aggravate P-SILI, through increases in transpulmonary pressure, either globally or limited to regional distribution. Therefore, the appropriate timing for the establishment of non-invasive ventilatory support can preclude effects on P-SILI and decrease of the respiratory drive can predict success (26).

The early recommendations at the beginning of the first wave were based on previous experiences in non-COVID patients and the consensus of experts. Some societies recommended starting non-invasive support when oxygen needs exceed FiO_2_ of 0.4, in addition to clinical criteria, mainly tachypnoea (27). The early Italian triage led to the identification of four patient categories: (a) green (SaO_2_ > 94%, respiratory rate (RR) < 20 breaths/min); (b) yellow (SaO_2_ < 94%, RR > 20 but responds to 10–15 L/min oxygen); (c) orange (SaO_2_ < 94%, RR > 20 but poor response to 10–15 L/min oxygen and requiring CPAP/NIV with very high FiO_2_); and (d) red (SaO_2_ < 94%, RR > 20 but poor response to 10–15 L/min oxygen, CPAP/NIV with very high FiO_2_ or presenting respiratory distress with PaO_2_/FiO_2_ < 200) and requiring endotracheal intubation and intensive care (28). In this classification, employed in a multicenter retrospective study (29), the indication to start NIV corresponded to the third degree of severity of the ARF (orange). The German position paper suggested starting O_2_ or HFOT when PaO_2_ ≤ 55 mm Hg and RR ≥ 30/min on room air (30). In the NHS guidelines, the criteria proposed for the initiation of CPAP and O_2_ were the inability to maintain SpO_2_ between 92 and 94% with an FiO_2_ between 0.4 and 0.6 (31). Some experts proposed two different scenarios for starting NIRS: Early start (PaO_2_: FiO_2_ < 300 or SpO_2_ < 93% on O_2_ > 5 L/min or SpO_2_ < 94% with FiO_2_ 40%) or late start (SpO_2_ < 92% under O_2_ at 15 L) (32). Regardless, definition of early start is not homogeneous, and there is scarce evidence to support it. García Pereña et al. retrospectively compared the use of early HFOT (in patients with PaO_2_/FiO_2_ > 100) vs. patients with PaO_2_/FiO_2_ < 100, finding significant differences regarding the rate of intubations (lower in the group with PaO_2_/FiO_2_ > 100), with mortality at the limit of significance (33). Deng et al. retrospectively compared mortality among elderly patients who received HFOT with a PaO_2_/FiO_2_ ratio between 200 and 300 (early) with another cohort with a ratio lower than 200 (late). Baseline conditions between both groups were similar and both mortality and complications were significantly lower in the group that received HFOT late (34). Obviously, both studies have the same limitation: in addition to being retrospective, there is a selection bias, since patients with “late initiation” represent a group that has previously failed to respond to conventional oxygen therapy, reflecting disease progression albeit treatment, while in the early group there are patients that may also respond to conventional oxygen therapy. Randomized, high-quality studies, are ongoing to define the effect of early HFOT in patients with ARDS secondary to COVID-19 (35).

On the other hand, randomized controlled studies not directed toward this endpoint also showed heterogeneity when determining the criteria for initiating NIRS. Thus, the Respiratory Support Recovery trial defined the clinical condition for randomization those patients with a need for FiO_2_ ≥ 0.4 and a peripheral SpO_2_ ≤ 94%(36), while the HENIVOT study (37) requires a PaO_2_/FiO_2_ of < 200 as the sole criterion for the initiation of the SRNI. It should be noted, as suggested by Winck and Scala, that the PaO_2_/FiO_2_ index may not reflect the severity of the exchange, as it does not take into account the baseline PaCO_2_ value, which is usually decreased in patients with ARF secondary to COVID-19 (38). More accurate seems to be the use of the alveolar-arterial oxygen gradient.

In addition to the opinions of experts, there may be another reason related to the technique of oxygen therapy administered in a Venturi effect mask. It was shown that the gas mixture from FiO_2_ of 0.4 can provide up to 50 L/min in the mask, so that in patients with high ventilatory drive that exceed these flow demands, the effective FiO_2_ in the mask may be lower (39).

## Non-invasive Support Modalities. Escalating Algorithms and the Role of Combined Therapies

Since the beginning of the pandemic, heterogeneous recommendations about the most preferred modality (HFOT, CPAP, NIV) appeared in the literature. Whereas some societies emphasized the need for early orotracheal intubation, others recommended a trial with non-invasive ventilatory support, with important differences in the first-line modality: most experts recommended HFOT, although others preferred treatment with positive pressure systems (mainly CPAP) and even with specific interfaces (helmet) (28, 30, 31).

The use of high nasal flow in non-COVID hypoxemic ARF is supported by high-quality controlled studies that show a decrease in mortality compared to conventional oxygen therapy and non-invasive ventilation, especially in patients with a PaO_2_/FiO_2_ ratio lower than 200. In addition, it is a better tolerated technique when compared with CPAP (17). Moreover, the distribution of tidal volume is more homogeneous than conventional oxygen therapy, protecting the lung against P-SILI (40). On the other side, the PEEP effect achieved is usually less than with true positive pressure systems and it should take into account that the combination of high FiO_2_ and low PEEP values maintained has long been associated with de-recruitment phenomena (resorption or denitrogenation atelectasis) in patients with acute lung injury (41). As maintained supraphysiological oxygen levels were associated with an increased mortality in a large, unselected multicenter cohort of critically ill patients (42), a close monitoring and later adjustment of inspired FiO_2_ in C ARDS patients seems adequate.

In clinical practice, in a survey that included responses from 502 units from 40 countries, high nasal flow was the most widely used NIRS modality (53%) in cases of mild-moderate ARF, followed by systems of positive pressure (47%) (25). In the same way, a study carried out in an ICU setting highlighted the heterogeneity of treatments between the different origins of the participants, although HFOT was the most used strategy (47%) followed by CPAP/NIV (26%) and early direct intubation (7%) (24). In fact, in an expert consensus based on the Delphi method, 97% of them agreed that HFOT can be considered as an alternative strategy for oxygen support before invasive mechanical ventilation, and should be used in patients who are unable to maintain SpO_2_ > 90% using oxygen delivery through a Venturi mask or may be used in patients with increasing oxygen requirement to avoid endotracheal intubation (43).

Regarding its efficacy, Demoule et al. in a retrospective study with data from the first 2 months of the pandemic, showed a lower intubation rate in the group that received high nasal flow compared to conventional oxygen therapy, although patients with this second group had more severe disease, with a higher rate of acute kidney failure and need for vasopressors (44). Similarly, Bonnet et al. also in a retrospective study, demonstrated an increase in ventilation-free days and a lower intubation rate in patients who received high nasal flow compared to those who received conventional oxygen therapy, but without any differences on mortality between the two groups (45).

The better tolerance and the lack of ICU beds during pandemic peaks have led to increased HFOT use outside the intensive care units, or in patients with do not intubate (DNI) orders (46). Medrinal et al. compared two cohorts of patients, a first group with DNI orders and a second group without therapeutic limitation. In the first group, mortality was 60% (lower in patients who received high nasal flow compared to those who received a miscellaneous group of therapies) while in the second group it was only 26%. In any case, whether HFOT was used in patients as a ceiling therapy or as a first line for *de novo* respiratory failure, it was associated with lower mortality. However, it is not clear whether the patients included in the study underwent sequential escalation treatment in case of failure of high nasal flow (47). In a small study in elderly patients, after adjustment, HFOT was associated with less mortality than conventional oxygen therapy (48).

There are few prospective studies comparing high nasal flow with other non-invasive support modalities. Grieco et al. in a randomized study (37), did not find any differences in mortality between the CPAP modalities with helmet and high nasal flow, although the intubation rate and days free from invasive ventilation were lower in the group that received CPAP. Finally, in the prospective study RS-RECOVERY (preprint), the use of conventional oxygen therapy vs. high nasal flow did not show differences in the composite endpoint intubation or mortality at 30 days (45.1 vs. 44%), while the CPAP group showed a lower incidence of such an endpoint (36).

The second therapeutic option for the treatment of ARF are the positive pressure devices, either CPAP or pressure support. The effect of expiratory positive pressure prevents alveolar collapse and improves ventilation-perfusion relationships and, ultimately, pulmonary gas exchange. The addition of pressure support can theoretically contribute to unloading inspiratory muscles. However, in hypoxemic ARF, the use of positive pressure systems, except for acute cardiogenic lung oedema, remains controversial. In fact, the expert consensus in the respiratory management ARF in COVID-19 recommended only NIV in presence of mixed respiratory failure (hypoxemia and hypercapnia) and in selected patients with increased work of breathing (43). The increased respiratory drive characteristic in COVID patients and their relatively preserved lung mechanics (compliance) can lead to high tidal volumes when using pressure support. High tidal volumes (>9.2 or 9.5 ml/kg) under NIV are associated with increased mortality (16, 49), probably related to “unprotective” mechanical ventilation. On the other hand, the use of high-quality pressure ventilators equipped with monitoring capabilities can help to monitor reliably and continuously the respiratory rate and the tidal volume, except for helmet interface use.

The early experiences of treatment with positive pressure have already demonstrated a superiority compared to conventional oxygen therapy in terms of the prevention of orotracheal intubation, even with a moderate sample size (50). Positive pressure systems have been recommended as the first line of non-invasive ventilatory support in COVID, especially in countries such as Italy or England (28, 31).

Among the positive pressure modes, the most widely used has been CPAP. In a meta-analysis that included 3,377 patients treated with positive pressure systems outside the Intensive Care Units, a total of 2,764 patients were treated with CPAP and 1,855 with helmet interface (22).

Treatment with positive pressure modes has been used in two different clinical situations: as preventive therapy for orotracheal intubation and as a rescue NIRS in patients with a therapeutic ceiling, mainly DNI orders. In an observational comparative study between both clinical situations, Walker et al. (51), demonstrated a mortality of 25% in the group of patients without DNI orders and 84% in the second, questioning whether CPAP offered an additional benefit in patients with therapeutic ceiling compared to conventional oxygen. In a single-center retrospective study, from 310 patients with ARF treated in the emergency department, 27 had DNI orders and were treated with CPAP, with the overall mortality at 88%. Finally, a UK multicenter study compared conventional oxygen therapy vs. CPAP as a ceiling of care in ward-based patients with COVID-19. Overall mortality showed no differences between the groups that received oxygen (75.6%) and CPAP (77.7%). Nearly 50% of patients who received CPAP chose to discontinue it (52). Despite being considered one of the best interfaces for delivering CPAP, Coppadoro et al. reported 75% of failure in DNI patients receiving CPAP through helmet outside ICUs (53).

In contrast, other studies reported lower rates of failure and mortality: in a prospective single-day study to describe the use of positive pressure systems outside the ICU, 85% received CPAP (68% with a helmet). Overall mortality was 25%, with a success rate of 60% (75% in patients without therapeutic limitation). The failure rate in patients with previous DNI orders was 52% (54). In the second wave, a UK study reported a 56% rate of survival in patients where CPAP was the ceiling of care. Interestingly, the mean time of CPAP use was 9 days (55). Similar results were reported by Aliberti et al. with a mortality of 55% (36/65) in patients with DNI orders using helmet CPAP (56). These discrepancies suggest that the success or failure of the technique is attributable to various aspects, such as the selection of patients, the experience of the team, or the specific protocols of each hospital, with differences in the starting criteria, the interface used, or the level of monitoring. Related to this latter issue, the value of respiratory intermediate care units has been demonstrated both as stepping down (patients transferred from the ICU) and stepping up methods. Matute-Villacís et al. reported 10% mortality in stepping down patients (most of them tracheostomized) and 25% in stepping up ones (57). When available, it would be important, even for selected patients with DNI orders, to organize medical units with basic monitoring capabilities and trained teams for delivering NIRS. In a Spanish survey, the number of existing intermediate care units in the Spanish Public Health System increased from 16 to 41 during the pandemic, bringing the increase in total beds from 112 to 525 (58).

Finally, measurements of activity and quality indicators should be implemented in each service providing NIRS outside ICU to acquire valuable data that may allow to enhance the provided care or determine if any improvement is needed. The final goal would be to use NIRS in selected DNI patients with higher survival probabilities, avoiding at the same time unnecessary extended dying processes in non-responders.

Regarding the efficacy to avoid intubation, in the previously mentioned meta-analysis, from the 75% of survivors in the group of patients who were candidates for intubation, 31% required IMV and 43% only SRNI (22). In a study including patients who were candidates for intubation and invasive ventilation but who could not receive such treatment due to the shortage in the context of massive influx of patients, intubation was avoided in 37% of patients, who were managed only with CPAP (59). Similar results (40% efficacy) were reported by Noeman-Ahmed et al. (60). Fairly better results were reported in a group of patients with moderate ARF (PaO_2_/FiO_2_ < 200 and RR < 30), with 85% of successful management exclusively with CPAP (61). A meta-analysis including more than 4,700 patients showed that CPAP and NIV were equally employed (48.4 vs. 46%). Interestingly, almost half of patients exposed to CPAP/NIV failed the non-invasive support trial and only half of failing cases were eligible for intubation. Finally, mortality was higher in patients treated with NIV (35.1%) than in patients treated with CPAP (22.2%), even though the number of failures was similar in each group (62).

Retrospective comparative studies between techniques of non-invasive support also offer heterogeneous results. The study by Franco et al. showed that there were no differences on mortality between patients who received NIV, CPAP, or HFOT, with mortality and the need for intubation being more related to the severity of respiratory failure (PaO_2_/FiO_2_ < 50), age, and number of comorbidities than with the type of support used (29). The proportion of NIRS failures was between 25 and 30% for the three modalities, despite the patients who were treated with NIV seeming to be in worse clinical conditions (more tachypnea and lower PaO_2_/FiO_2_ ratio). Interestingly, in all the hospitals that participated in the study, patients were treated in monitored areas by skilled teams. A study conducted in Ireland with a similar design compared oxygen therapy, positive pressure, and HFOT: an improvement in arterial blood gases was documented mainly in patients transitioned from oxygen to CPAP but without differences on mortality both in patients with and without DNI order (63).

In a matched retrospective of COVID-19 patients admitted to the ICU, the four therapeutic supportive therapies (oxygen therapy, high nasal flow, non-invasive ventilation, and direct intubation) were compared. The group with the highest mortality received non-invasive ventilation (64).

There are a few prospective randomized controlled studies comparing different non-invasive support modalities. Grieco et al. (37), in the HENIVOT study, randomized 110 patients to receive support therapy with HFOT or helmet CPAP. The primary endpoint was the number of days free of respiratory support at day 28. The nine secondary endpoints were related to need for intubation, mortality, ICU stay, and number of days free of invasive ventilation. Among the nine secondary endpoints, only the intubation rate and number of days free of invasive ventilation achieved statistical significance, both favoring the group of helmet CPAP.

The RS-Recovery trial (36) is a three-arm randomized controlled trial on three non-invasive respiratory strategies (conventional oxygen therapy, high flow, and CPAP). The primary outcome was a composite of tracheal intubation or mortality within 30 days. 1,272 patients were randomized. The need for tracheal intubation or mortality within 30 days was lower in the CPAP group (35%) whereas no differences were found in HFOT and conventional oxygen therapy group (44.4 and 45.1% respectively). Interestingly, all interfaces were permitted in the CPAP group, not exclusively helmet. Some crossovers between groups should be noted as a limitation, although they may have favored the conventional oxygen group.

In clinical practice, however, it is not common to find patients with a pure ventilatory support strategy throughout the course of the disease. Patients often receive a variety of supportive treatments, escalating in case of a lack of response or in combination. In the first setting, positive pressure therapy has also been recommended in case of insufficient response to high flow (30, 38). In this regard, it should be noted that a group of English experts considered the use of NIMV as inappropriate in case of failure of the first line of treatment, recommending direct intubation (65).

Both scenarios were retrospectively studied by Colaianni et al. (66) in a clinical study conducted under a careful algorithm for managing ARF in COVID patients. The first step was HFOT and prone position. In case of failure, a CPAP trial, combined with periods of HFOT, was initiated. The first step had a failure rate of 10/65, but mainly due to CPAP intolerance. In the second group (HFOT + CPAP) the failure rate was 20/48. Mortality in intubated patients was 55%. Of note, combination of modalities is not uncommon in clinical practice, especially pauses in CPAP/NIV therapy using HFOT, for example for feeding breaks (29).

Finally, prone position in non-intubated patients has been a complementary strategy for managing COVID patients with ARF. In patients who are intubated and have moderate to severe acute respiratory distress syndrome, prone positioning is an effective intervention to improve oxygenation and reduce mortality, while improving ventilation in dependent lung areas. It is recommended in guidelines for patients with a PaO_2_/FiO_2_ ratio < 150, in sessions of 16 h/day (67). Awake prone positioning has been associated with improved oxygenation in observational studies of non-intubated patients with acute respiratory distress syndrome (68) and, more recently, it has been demonstrated that it is feasible in patients with COVID-19, with improvements in blood oxygenation that are maintained after re-supination in about half of patients (69). The expert consensus stated that awake self-proning may improve oxygenation when used in patients with C-ARDS requiring supplemental oxygen to maintain oxygen saturation > 90% (43).

The APRONOX study (70), compared outcomes of patients with various sources of oxygen therapy (low-flow, high-flow, and reservoir mask) who underwent prone sessions of at least 2 h duration. The total mean duration of the prone was 12 h during the entire hospital stay and the SpO_2_/FiO_2_ ratio increased significantly after the prone sessions (from 183 to 212). There were also significant differences in the proportion of intubations (23% in the prone group, 40% in the supine group). Mortality in intubated patients was close to 70%.

Ehrmann et al. (71), in a meta-trial that included patients from six different trials, compared the outcome of 1,126 patients randomized to high flow and prone position or to high flow and standard treatment. Patients with a PaO_2_/FiO_2_ lower than 300 were included, although the mean PaO_2_/FiO_2_ in both groups at the time of randomization was around 150. Prone time was variable, with a mean of 5.6 h, but with wide variation among participating countries (from 1.6 to more than 8 h). The composite endpoint (treatment failure or death) was significantly lower in the high-flow and prone group. To avoid treatment failure, a NNT of 15 was required. 28-day mortality was not statistically significant globally or in the group of patients who failed in both groups, which shows that the prone test did not worsen the prognosis of patients who failed. Finally, patients in the prone group were more likely to be released from high flow therapy than the control group.

Despite the beneficial effects on blood oxygenation of awake proning, a proportion of patients, which could be up to 60%, do not tolerate it (69, 72, 73). A variant of postural treatment (Rodin's thinker) has recently been proposed, with the patient sitting on a chair and rest their chest on a flat, elevated surface (semi-prone position). Coppo et al. reported a significant improvement in blood oxygenation in 25 patients with this postural treatment. After re-supination, the blood oxygenation was better than the baseline values (74).

Table 1 summarizes the main studies about NIRS, with emphasis in the NIRS starting criteria, type of support, and results.

**Table 1:** Summary of the main studies about NIRS, with emphasis in the NIRS starting criteria, type of support, and main results.

**References**	***N***	**Design**	**Criteria for starting non-invasive support**	**Type of support or intervention**	**Environment/DNI status**	**Main results**
Perkins et al. (36)	1,272	RCT	SpO_2_ > 94 on FiO_2_ 0.4	CPAP (*m* = 380) HFNC (*n* = 417) COT (*n* = 475)	Not stated/full treatment (no ceiling)	CPAP associated with less mortality and intubation than COT (36 vs. 44%). No advantage of HFNC
Griecoet al. (37)	110	RCT	PaO_2_/FiO_2_ < 200. Non-hypercapnic	Helmet CPAP vs. HFNC	ICU/no ceiling	No differences in 28 d mortality. Helmet CPAP associated with less intubation than HFNC (30 vs. 51%)
Franco et al. (29)	670	Retrospective observational	SaO_2_ < 94%, poor response to 10–15 L/min oxygen.	HFNC CPAP NIV	Pulmonary Ward (4% with DNI orders)	30-day mortality HFNC: 16% CPAP 30% NIV 30%/ ETI rate: HFNC 27% CPAP 25% NIV 28%
Aliberti et al. (56)	157	Retrospective observational	PaO_2_/FiO_2_ < 300 with O_2_ at (FIO_2_ of at least 0.50) or reservoir mask.	Helmet CPAP	High dependency Unit/41% DNI orders	CPAP failure was observed CPAP failure 45%, 21% ETI (of them, 26% died), 22% dead in HDU. CPAP failure associated with IL-6 levels, and severity scores
Oranger et al. (50)	66	Retrospective observational	SpO_2_ < 92% with O_2_ 6 lx'	CPAP vs. COT	Pulmonary ward/12% DNI orders	57% failure prealgorithm, reduced to 23% post algorithm
Demouleet al. (44)	379	Retrospective observational	RR > 25 Need for O_2_ ≥ 3 l/min for Spo2 ≥ 92%	HFOT vs. COT	ICU/no ceiling	Higher baseline severity in COT group Intubation rate 56% in HFOT group vs. 75% in COT group
Bonnet et al. (45)	138	Retrospective observational	RR > 25 Need for O_2_ ≥ 3 l/min for Spo2 ≥ 92%	HFOT vs. COT	IC/no ceiling	Intubation rate 51% in HFOT group vs. 74 % in COT group. No differences on mortality. Higher severity in the HFOT group at ICU admission (higher RR and O_2_ needs)
Medrinal et al. (47)	400	Retrospective observational	PaO_2_/FiO_2_ < 300 or SpO_2_ < 94% with at least O_2_ 10 L/min	Multiple therapies (COT, HFOT, CPAP, NIV, and combinations)	ICU/Intermediate care unit/32.5% DNI orders	Mortality: 60% in the group with DNI orders, 26% in full treatment group. Lower mortality with HFOT in DNI orders.
Walker et al. (51)	294	Retrospective observational	SpO_2_ < 94% with FiO_2_ 0.4	CPAP vs. COT	ICU and ward/DNI orders 53.4%	Mortality: 84% in the group with DNI orders, 25% in full treatment group.
Bradley et al. (52)	479	Retrospective observational	Need for FiO_2_ ≥ 0.4. Clinical frailty score < 6	CPAP vs. COT	Ward(100% DNI orders)	No differences on mortality (75 % in COT group, 77 % in CPAP)
Coppadoro t al. (53)	306	Retrospective observational	Reservoir mask and: SpO_2_ < 93% or RR > 24.	Helmet CPAP	Ward (42% DNI orders)	Helmet CPAP was successful in 28% DNI order group and in 69% full treatment group
Gough et al. (63)	164	Retrospective observational	>4L/min oxygen to maintain SpO_2_ > 92%	CPAP = 85 HFOT = 32 COT = 47	Ward (33.5%DNI orders)	Mortality 56% in DNI group without differences on NIRS techniques. No differences on IMV ratio between techniques in full treatment group
Perez Nieto et al. (70)	827	Retrospective observational	SpO_2_ < 94 % (room air)	Awake proning vs. no proning	ICU/Ward	Lower intubation and mortality rates in awake proning (both matched and non-matched models). 70% mortality in intubated patients.

## Evaluation of the Response to SRNI

Early evaluation of the established non-invasive support modality seems to be of the utmost importance when deciding whether to continue with the same therapeutic approach, change the modality, or proceed with orotracheal intubation.

In the use of HFOT, one of the most widely used indices in clinical practice is the ROX (SpO_2_/FiO_2_: RR) at 2, 6, and 12 h after starting treatment (75). An increase in this index has been associated with patient improvement. The cut-off point accepted in the pre-COVID era in patients with pneumonia and hypoxemic ARF (75) was 4.88 in the ranges described (rates higher than 4.88 were associated with treatment success).

Specifically in COVID patients, Chandel et al. demonstrated that a ROX index > 3.0 at 2, 6, and 12 h after initiation of HFOT was 85.3% sensitive for identifying HFOT success (76). On the contrary, Zucman et al. determined that the most sensitive cut-off point for intubation risk was 5.37 at 4 h (77). Finally, in patients with whom high nasal flow was indicated outside the ICU, Vega et al. determined that the value with the highest sensitivity was 5.9, while the classic value of 4.88 was not sufficiently discriminating (78). However, regardless of the specific cut-off point, it seems more reasonable to monitor the trend of the ROX index throughout treatment, as proposed by Xia et al. who demonstrated that the trend to decrease in the ROX index and the increase in the RR over 3 days were predictors of failure (79).

Other authors proposed only the change in respiratory rate as a predictor of HFOT success or failure. Blez et al. demonstrated a discriminant power of the change in RR 30 min after starting the treatment similar to that of the ROX index (80).

In positive pressure treatment, Amati et al. studied the response in the recruitment in a group of patients who were shifted to CPAP and helmet, with PEEP values up to 15 cm H2O. Of the 34 patients included in the study, only nine had a complete response and 17 a partial response. The parameters proposed to consider a response as complete (all of them had to be met) were a 20% decrease in the alveolar-arterial gradient, a decrease in respiratory rate with respect to baseline, an increase in Spo2, and good clinical and hemodynamic tolerance (81). Aliberti et al. defined lung recruitability during helmet CPAP treatment as an increase of PaO_2_/FIO_2_ ratio of at least 30% from oxygen therapy (baseline) to CPAP treatment (within 6 h) (56). This endpoint was achieved only in 52% of the study population.

Similar short-term criteria were described by De Vita et al. as predictors of CPAP treatment failure (in addition to age and lactate level). While in patients with CPAP failure, the improvement in PaO_2_ was 19%, in CPAP success it was 59% (82).

The HACOR score (Heart rate, Acidosis, Consciousness level, Oxygenation, and Respiratory rate) has been proposed as a bedside tool for predicting NIV failure (83). It has also been explored as a predictive score for CPAP failure in a multicenter study. Although the performance was quite good (82%), it was similar to PaO_2_/FiO_2_ ratio (81.25%) (84).

In an interesting study with continuous measurement of esophageal pressure as a surrogate of patient's inspiratory effort, Coppola et al. demonstrated that the early predictors of failure (measured on the first day of treatment) under CPAP or pressure support treatment were the PaO_2_/FiO_2_ ratio, the intensity of changes in esophageal pressure, and the total stress lung. This last concept, which was the only independent factor related to failure in the multivariate analysis, is equivalent to the total transpulmonary pressure, and includes concepts such as applied pressure support, changes in esophageal pressure, or set PEEP value (85).

## The Duration of Non-invasive Support. Failure Criteria. How to Deescalate

The duration of NIRS in COVID patients seems clearly longer than in non-COVID patients, but with huge variability. In the meta-analysis by Cammarota et al. (22) the mean time of non-invasive support (CPAP-NIV) until orotracheal intubation in patients with NIRS failure ranged between 72 and 137 h. In responders, the mean time of total duration of NIRS ranged between 2 and 12 days.

This long NIRS time may increase the probability of late failure, with a worsening of the prognosis if intubation is required. This point has been the subject of research in few studies. In an observational study, Boscolo et al. determined that the ventilation time prior to admission to the ICU was one of the determining factors of mortality in patients in whom NIV failed. Although there were no significant differences between patients who were directly intubated and those who underwent failed NIV trial prior to intubation, in patients with a duration of ventilation >48 h outside the ICU, the authors found a significant increase in mortality (86). Similarly, Vaschetto et al. determined that CPAP use time ≥ 3 days was an independent predictor of mortality in the event of CPAP failure and intubation (87).

Given these data, it seems especially important to closely monitor patients under NIRS who are treated for more than 72 h with any supportive therapy. In the event of late deterioration in respiratory conditions in these patients, orotracheal intubation and invasive mechanical ventilation should be considered immediately. In addition to the classic criteria for invasive ventilation (hemodynamic instability, decreased level of consciousness, appearance of signs of muscle fatigue, or development of unmanageable tracheal secretions), predefined respiratory conditions for intubation should be protocolized, especially in late failure. For example, Aliberti et al. proposed a combination of major and minor criteria for considering intubation (at least 1 major or at least two minor criteria lasting for ≥1 h). The reduction of ≥30% of basal PaO_2_/FIO_2_ ratio, the PaO_2_/FIO_2_ ratio < 100 and the increase of arterial carbon dioxide tension if basal arterial carbon dioxide tension was ≥40 mmHg, and oxygen saturation measured by pulse oximetry (SpO_2_) < 90% a are some of the minor criteria (56). The HENIVOT study defined failure and need for invasive ventilation when two or more were present: the oxygenation worsening was defined as oxygenation and/or SpO_2_ below 90% for more than 5 min (37). It is also important to rule out pulmonary embolisms as a potential cause of acute oxygenation alterations, the incidence of which has been shown to be higher in COVID patients under ventilatory support (88).

In summary, it would be cautious to consider orotracheal intubation in those patient candidates who after 48-72 h of NIRS do not present significant clinical improvement, as well as in those patients with acute worsening of a previously stable situation, or with highly compromised respiratory conditions (PaO_2_/FiO_2_ < 100).

Finally, prolonged treatment times with NIRS may require progressive support withdrawal. Up to now, there have been no definite results about the ideal method. In patients using HFOT, the ongoing SLOWH study protocol proposes the comparison between three branches for the withdrawal of high nasal flow (low FiO_2_, low flow, or simultaneous) (89).

For CPAP users, the model proposed and standardized across the three hospitals that participated in the study was as follows: patients who did not show signs of respiratory distress (e.g., respiratory rate < 25 breaths·min^−1^) and maintained a SpO_2_ > 94% with a FIO_2_ < 50% and a PEEP ≤ 5 cmH2O underwent a weaning trial. Patients maintaining a PaO_2_/FIO_2_ ratio > 250 on Venturi mask with a FIO_2_ < 40% for at least 24 h were considered successfully weaned from helmet CPAP (56). In the HENIVOT study, weaning was performed by reducing positive end-expiratory pressure and pressure support to 8 cmH2O. If the patient maintained SpO_2_ ≥ 92% and respiratory rate equal to or lower than 25 breaths/min for 30 min, non-invasive ventilation was interrupted (37).

## Conclusions

The use of non-invasive support, especially in situations of high simultaneous influx of critical patients, helps to avoid intubations and invasive mechanical ventilation in COVID patients. The decision for starting NIRS is a combination of oxygenation derangement (PaO_2_/FiO_2_ ratio, alveolar-arterial gradient) and clinical signs (tachypnea and inspiratory effort). Albeit scarce, the few high-quality randomized controlled studies have shown an advantage of Continuous positive airway pressure over other respiratory support techniques. In addition, HFOT plus prone position is a promising first step approach, and for some milder respiratory failure, HFOT alone may be an acceptable approach over COT.

For any kind of respiratory support employed, it is mandatory to monitor the efficacy in a short time frame. In the absence of response, prompt orotracheal intubation and invasive ventilation needs to be considered, if the patient is a candidate for full therapy. If the condition of the patient under NIRS remains stationary after 48-72 h, orotracheal intubation should also be considered. Not all the patients may be candidates for invasive ventilation. For those patients with DNI orders who receive non-invasive ventilatory support, high mortality can be expected. It should be taken into account while starting or maintaining potentially futile treatments (in cases without response) that are not free from secondary effects and may pose relevant discomfort in dying patients.

## Author Contributions

All authors listed have made a substantial, direct, and intellectual contribution to the work and approved it for publication.

## Conflict of Interest

The authors declare that the research was conducted in the absence of any commercial or financial relationships that could be construed as a potential conflict of interest.

## Publisher's Note

All claims expressed in this article are solely those of the authors and do not necessarily represent those of their affiliated organizations, or those of the publisher, the editors and the reviewers. Any product that may be evaluated in this article, or claim that may be made by its manufacturer, is not guaranteed or endorsed by the publisher.
